# Differential methylation as a diagnostic biomarker of rare renal diseases: a systematic review

**DOI:** 10.1186/s12882-019-1517-5

**Published:** 2019-08-16

**Authors:** Katie Kerr, Helen McAneney, Cheryl Flanagan, Alexander P. Maxwell, Amy Jayne McKnight

**Affiliations:** 1 Centre for Public Health, Queen’s University Belfast, c/o Regional Genetics Centre, Level A, Tower Block, Belfast City Hospital, Lisburn Road, BT9 7AB Belfast, Northern Ireland; 2 100,000 Genomes Project Team, Belfast Health and Social Care Trust, Belfast, Northern Ireland; 3 Regional Nephrology Unit, Belfast City Hospital, Belfast, Northern Ireland

**Keywords:** Methylation, Rare disease, Biomarker, Diagnosis, Renal

## Abstract

**Background:**

The challenges in diagnosis of rare renal conditions can negatively impact patient prognosis, quality of life and result in significant healthcare costs. Differential methylation is emerging as an important biomarker for rare diseases and should be evaluated for rare renal conditions.

**Methods:**

A comprehensive systematic review of methylation and rare renal disorders was conducted by searching the electronic databases MEDLINE, EMBASE, PubMed, Cochrane Library, alongside grey literature from GreyLit and OpenGrey databases, for publications published before September 2018. Additionally, the reference lists of the included papers were searched. Data was extracted and appraised including the primary focus, measurement and methodological rigour of the source. Eligibility criteria were adapted using the inclusion criteria from ‘The 100,000 Genomes Project’ and The National Registry of Rare Kidney Diseases, with additional focus on methylation.

**Results:**

Thirteen full text articles were included in the review. Diseases analysed for differential methylation included glomerular disease, IgA nephropathy, ADPKD, rare causes of proteinuria, congenital renal agenesis, and membranous nephropathy.

**Conclusions:**

Differential methylation has been observed for several rare renal diseases, highlighting its potential for improving molecular characterisation of these disorders. Further investigation of methylation following a standardised reporting structure is necessary to improve research quality. Multi-omic data will provide insights for improved diagnosis, prognosis and support for individuals living and working with rare renal diseases.

**Electronic supplementary material:**

The online version of this article (10.1186/s12882-019-1517-5) contains supplementary material, which is available to authorized users.

## Background

Whilst rare diseases are uncommon at the individual level, cumulatively they represent a significant public health problem with approximately 350 million people suffering worldwide [1]. The definition of a rare disease varies between continents; the European Commission classifies a rare disease as one which affects less than five people in 10,000, whilst the American definition states rare diseases affect fewer than 200,000 people [1]. An underlying genetic cause is suspected in 80% of rare conditions, with 50% of these diseases occurring in children. Two in five patients with rare diseases describe struggling to obtain a timely accurate diagnosis which can be detrimental to each individual’s quality and length of life [2]. Interpretation of massive amounts of genetic information generated by large scale sequencing efforts remains a challenge; but despite this, these efforts are significantly improving the speed and accuracy of rare disease diagnosis [3]. Additionally, rationalising data generated by multi-omic approaches could provide new insights into molecular profiles for rare diseases.

More than 100 rare renal disorders have been reported [4, 5]. Similar to many other rare diseases, clinical diagnosis of rare renal diseases may be challenging with limited clinically relevant biomarkers, significant phenotypic variability, poor understanding of the disease pathogenesis and a lack of appropriate therapies. For example, diagnosis of IgA nephropathy (IgAN) involves a renal biopsy to confirm diagnosis which is an invasive and relatively expensive procedure [6]; diagnosis of polycystic kidney disease (PKD) utilises ultrasound scanning for diagnosis, which is insufficiently sensitive to detect the earliest stages of PKD even though earlier detection may improve the patient’s prognosis [7]. The development of a panel of cost-efficient, sensitive and accurate biomarkers of rare kidney disease which can be detected non-invasively would greatly aid rare renal disease diagnosis. A biomarker is defined as “a characteristic that is objectively measured and evaluated as an indicator of normal biological processes, pathogenic processes, or pharmacologic responses to a therapeutic intervention” [8]. At present, biomarkers to identify renal dysfunction are sub-optimal and often based on inexact biochemical markers where half of ‘normal’ kidney function is lost before kidney disease is identified. Additionally it is important to note that kidney dysfunction (associated with gene variants or complex chromosome abnormalities) can have widespread impacts on other organ systems [5]. Therefore, multi-centre studies are required to establish disease-based cohorts for rare renal disease with consistent biological sample collection, harmonised biomarker measurements, effective data sharing, and compatible analytics across all data.

Unravelling the genetic basis of rare kidney disorders has been facilitated by the establishment of rare renal disease biobanks and registries such as The PodoNet Registry for congenital and steroid resistant nephrotic syndrome [9], the UK National Registry of Rare Kidney Diseases (RaDaR) [10], and the development of focused international professional multidisciplinary teams such as the ERA-EDTA Working Group on Inherited Kidney Disorders [11]. Current research to improve knowledge of disease is moving beyond simple changes to the DNA sequence to utilising integrated multi-omic strategies to improve clinical diagnosis [12]. There are 517 omics currently described [13], with transcriptomics (RNA-based), epigenomics (DNA and RNA based non sequence level modifications), and proteomics being the primary research tools for rare renal diseases at present. Integrated molecular and clinical approaches are increasingly being employed to provide functional evidence for pathogenicity of SNPs (single nucleotide polymorphisms) and to deliver novel disease insights [14–17].

Methylation, the addition of a chemical methyl group via DNA methyl transferases, is a key epigenomic feature affecting gene expression. Methylation predominately occurs in CpG dinucleotides (though it can occur elsewhere) where the methyl group is added to the fifth carbon of the cytosine forming 5-methylcytosine (5mc) [18]. Large-scale methylome-wide studies have allowed better understanding of DNA methylation and health-related outcomes, for example using Illumina’s Infinium MethylationEPIC array, which quantitatively and cost effectively interrogates approximately 850,000 features [19], or deep whole-genome bisulfite sequencing of genomic DNA [20]. Evidence is growing that methylation risk scores (analogous to genetic risk scores) may be constructed for multiple health outcomes [21]. Methylation is largely considered be a transcriptional repressor with roles in genomic imprinting [22], X Chromosome inactivation [23], repression of repetitive elements [24], aging [25] and tissue specific gene expression [26]. The location of the methylated sites affects function, for example methylation within the gene body rather than the classically thought of transcriptional start sites may have a role in transcriptional activation [27].

Aberrant methylation is implicated in multiple disorders affecting a range of organ systems. These include, but are not limited to, vascular complications in type 2 diabetes despite good glycaemic control [28], several cancers [29], clinical heterogeneity in Alzheimer’s disease [30], pre-natal and early childhood neurodevelopmental disease [31], development of chronic kidney disease [32, 33], and differential methylation has even been implicated by a number of studies as potentially impacting kidney transplant outcomes through alloimmune response and ischemia–reperfusion injury [34]. The crucial role of DNA methylation in monoallelic imprinting is now evident from disorders of methylation, when aberrant methylation has detrimental effects on development, as is often the case in Prader–Willi syndrome, Silver–Russell syndrome, Beckwith–Wiedemann syndrome and Type Ib pseudohypoparathyroidism [35, 36]. The sophistication of epigenetic tools for disease characterisation continues to improve, such as the development of the EpiSign assay which can aid the diagnosis of 19 often difficult to identify disorders, including Angelman Syndrome, Prader-Willi syndrome and Beckwith-Wiedemann syndrome [37]. DNA methylation can be influenced by inherited (genetic) and acquired throughout life (somatic; environmental) factors, creating changes that may be short-acting, exist long-term in an individual, and / or demonstrate transgenerational inheritance. Several recent multi-centre papers confirm socioeconomic experiences across the life course impact peripheral blood-derived methylation, persisting from pre-birth, throughout childhood, to later adult life [38–41].

### Aims and objectives

This review summarises current evidence that exists for differential methylation in rare renal diseases by:

1. Identifying rare renal diseases that have been analysed for differential methylation.
2. Determining how differential methylation has been measured and in which genomic regions.
3. Discussing the potential for differential methylation as a diagnostic biomarker for rare renal diseases.

## Methods

This review was designed using the Preferred Reporting Items for Systematic Reviews and Meta-Analyses (PRISMA) systematic review checklist (Additional file 1) [42].

### Eligibility criteria

Quantitative articles written in English and published before September 2018 were included if they were relevant to non-cancerous rare renal diseases which appear in the inclusion criteria from ‘The 100,000 Genomes Project’, [43] and were directly relevant to aberrant DNA methylation. Studies of kidney cancer and differential methylation was excluded as this has been previously reviewed [44]. Further articles were included if they were relevant to a condition that appeared on the inclusion criteria of the Registry of Rare Renal Diseases (RaDaR). It should be noted that although Autosomal Dominant Polycystic Kidney Disease (ADPKD) is not classed as a rare disease, the acquisition of a second mutation that causes the disease (a two-hit hypothesis) is a rare phenomenon and so it meets ‘The 100,000 Genomes Project’ inclusion criteria and that of this review. However, any rare renal disease included in RaDaR that was explicitly excluded in ‘The 100,000 Genomes Project’ was not included in search terms (e.g. Shiga toxin associated atypical-HUS). Of the RaDaR inclusion criteria, rare diseases that have renal involvement, but which are not primarily classed as a renal disease were also excluded. These were; vasculitis, tuberous sclerosis, retroperitoneal fibrosis, pure red cell aplasia, hyperoxaluria, HNF-1B mutations, fibromuscular dysplasia, Fabry disease, EAST syndrome and calciphylaxis.

### Information sources and search terms

Four electronic databases were searched for identification of primary sources: MEDLINE via Ovid, EMBASE via Ovid, PubMed and Cochrane Library. It is worth noting that PubMed was searched in addition to MEDLINE, as MEDLINE is a subset of PubMed which allows more specific searching but can return different results [45]. A search was also conducted of grey literature using the databases GreyLit and OpenGrey. Reference lists of included papers were also screened for further sources. Finally, websites genomeweb (https://www.genomeweb.com/) and Epigenesys (https://www.epigenesys.eu/en/) were searched for relevant articles. Search terms were created using the Population, Intervention, Comparison, Outcome (PICO) framework [46], primarily for use in MEDLINE and adapted for search in other databases, (see Additional file 2: Table S1). That is, the population of interest were patients or models of the rare renal diseases, interventions were varying measurements of differential methylation, comparisons were the individuals/samples without these rare renal diseases, and the possible outcomes were identification of differential methylation elucidating any potential applications to improve patient health or quality of life.

### Study selection, data extraction and critical appraisal

Database searches were last conducted on the 17th September 2018. Duplicates were removed, and the remaining papers were screened through analysis of their titles, abstracts and keywords for relevance. If relevant, the papers were then further screened by reading the full text. References and forward citations were also screened of the remaining papers to look for any further relevant papers. Data was extracted, (in duplicate by two independent personnel) and sources were critically appraised using a customised form modelled on the Joanna Briggs Institute Critical Appraisal tools [47], templates of which are available in (Additional file 2: Tables S2 and S3). Methodological rigour was scored as being weak, moderate, or strong with decisions based on choice of appropriate controls, how study limitations were addressed and the use of appropriate statistical analysis.

## Results

**Fig. 1:**
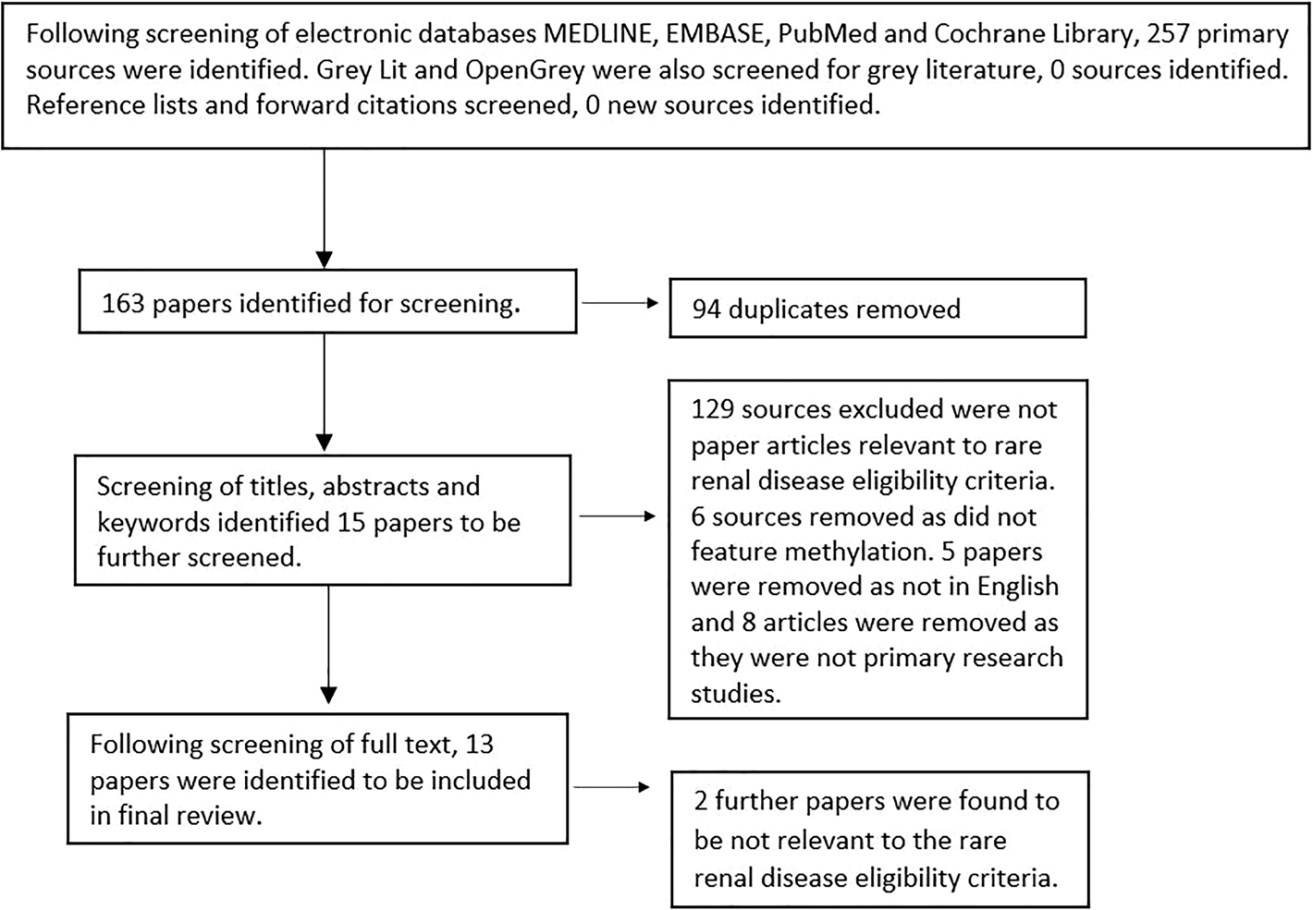
Illustration of search strategy including databases searched and screening methods modelled on the PRISMA flow diagram

Sources initially identified from each database were as follows, MEDLINE *n* = 58, EMBASE *n* = 136, PubMed *n* = 62, Cochrane Library n = 1, GreyLit *n* = 0, OpenGrey *n* = 0. Following title and abstract screening, 94 duplicates were removed and 15 papers were identified for full text screening. No further studies were identified from searching genomeweb and Epigenesys publication databases. Finally, 13 texts were included in the full review, (Fig. 1), with the characteristics of each source summarised from the completed data extraction forms, (Additional file 2: Table S4). This included study aims, design information, methylation measurement, methodological rigour and key results. Outcomes, methylation measurement and participant information are briefly summarised (Table 1). Of the texts included, 12 were case-control studies and one was a case report. Reference lists of these papers and forward citations were also screened but no further sources were identified. Of the 13 articles included in this review, three originated from South Korea, two from China, one from Italy, one from Canada, three from the United States of America and three from Japan. Methodological rigour was assessed as weak for all studies, primarily based on lack of description dealing with limitations, such as regression to identify confounding factors, lack of appropriate matching of cases to controls on the basis of gender, age or ethnicity, and often a lack of detail on experimental controls, statistical analysis or animal model strains used.

**Table 1:** Overview of study characteristics, ordered alphabetically by rare renal disease type

First author, year and rare kidney disease featured.	Methylation measurement method	Participant information.	Outcome
Fujino, Takayuki. 2016 Disease: Membranous Nephropathy	ChIP^a^ assays and H3K4me3^b^ localisation.	Renal biopsies from patients (n = 6) diagnosed with nephrotic syndrome caused by membranous nephropathy compared to controls with other causes of nephrotic syndrome. Two additional control comparisons were participants with microhematuria but no glomerulonephropathy (n = 3) and a single age matched healthy control. Also murine models with induced proteinuria.	The association of increased H3K4me3 and cathepsin L as well as decreased synaptopodin levels and proteinuria in membranous nephropathy.
Hayashi, K. 2014 Disease: Proteinuria	Micro-array based genome wide DNA^c^methylation profiling system, MSP^d^ and bisulphite sequencing.	Murine models of proteinuric disease, including FSGS^e^ (*n* = 11), minimal change disease (n = 10), diabetic nephropathy (n = 9) and normal controls (*n* = 9). Human renal biopsies from the same proteinuric diseases, number not stated.	Elucidation of a potential novel therapeutic target of proteinuria, the gene *KLF4*, including investigation of promoter CpG methylation.
Hayashi, K. 2015 Disease: Proteinuria	Bisulphite treatment of DNA and MSP.	Murine models of adriamycin nephropathy, (n = 5 in each treatment group). Samples from patients with proteinuric glomerular diseases including FSGS (n = 8), minimal change disease (n = 9), diabetic nephropathy (*n* = 8) and normal controls (n = 8). Immortal human podocyte cell lines.	Identification of *KLF4* as a potential therapeutic target of proteinuria and angiotensin receptor blockers as a treatment which exerts effects on methylation.
Ito, Y. 2017 Disease: Proteinuria	ChIP assays of histone methylation.	Human embryonic kidney cell lines as well as murine and zebrafish models of proteinuria.	The role of *WHSC1L1-L1* in epigenetically modifying the expression of nephrin, with implications for both congenital nephrotic syndrome (rare) and acquired nephrotic syndrome (non-rare).
Jin, M. 2014 Disease: Congenital renal agenesis	Reduced representation bisulphite sequencing to allow analysis of differentially methylated regions.	Chinese female monozygotic twins discordant for congenital renal agenesis.	Genomic/epigenomic changes, including methylation, which may be correlated with congenital renal agenesis in discordant monozygotic twins.
Li, LX. 2017 Disease: ADPKD^*f*^	ChIP with anti-H3K4me2^*g*^ antibodies and anti-SMYD2 antibodies. Methylation sites localised using a flag-tagged protein.	Double conditional knockout of *Pkd1* and *Smyd2* in murine models of ADPKD (*n* = 12) compared to single knockout of *Pkd1* (*n* = 14). Treatment of mice (*n* = 12) of ADPKD (*Pkd1* knockouts) with AZ505 compared to DMSO^*h*^ injected controls (n = 12) and in conditional *Pkd1* knockouts (n = 14) compared again to DMSO injected controls (n = 14). Human ADPKD cells were also utilised and compared to normal kidney cells.	SMYD2’s potential role in ADKPD cyst formation, including differential methylation.
Majumder, Syamantak. 2018. Disease: Proteinuria	Immunohistochemical staining, RT-qPCR^*i*^ and ChIP assays of H3K27me3^*j*^.	Murine models with induced glomerular injury compared to controls. Kidney samples of human participants with diabetic glomerulosclerosis (n = 12) compared to age matched healthy controls (n = 12) and FSGS (n = 10) compared to non-FSGS tissue biopsies taken at the time of kidney transplantation (n = 9).	Reduced H3K27me3 and subsequent upregulation of the Notch pathway as a contributor to albuminuria in glomerular disease.
Qi, S. 2012 Disease: IgAN^*k*^	ChIP microarray and real time quantitative MSP.	PBMCs^*l*^ from IgAN patients (*n* = 15) and healthy controls (n = 15).	Identification of H3K4me3 as a potential contributor to IgAN.
Sallustio, F. 2016 Disease: IgAN	Whole genome microarray analysis of CD4+ T cells, followed by pyrosequencing for validation.	Renal biopsies from IgAN patients (*n* = 24) and normal controls (n = 24).	Differential methylation in CD4+ T-cells as a potential contributor to IgAN pathogenesis.
Sui, WG. 2014 Disease: Membranous nephropathy	ChIP-sequencing of H3K9me3^*m*^ followed by Model-based Analysis of ChIP-sequencing which identified enriched H3K9me3 peaks.	PBMCs from membranous nephropathy patients (n = 10) and healthy controls (*n* = 10).	Identification of H3K9me3 alterations, including differential methylation, as a potential contributor to membranous nephropathy pathogenesis and a potential biomarker.
Sun, Q. 2015 Disease: IgAN	MSP of bisulphite treated *Cosmc* gene promoter regions.	PBMCs from paediatric patients with IgAN (*n* = 26), other renal diseases (n = 11) and healthy control children (n = 13).	Differential methylation in the *Cosmc* gene as a potential contributor to aberrantly glycosylated IgA1 in IgAN patients.
Woo, YM. 2014 Disease: ADPKD	MIRA-seq^*n*^ and ChIP-qPCR.	Cystic renal cortex samples from ADPKD patients (n = 3) and non-ADPKD samples from renal cell carcinoma patients (*n* = 3) used as a normal control. Madin-Darby Canine Kidney cells also used.	Differential methylation in ADPKD cyst formation and the role of methylation inhibitors in repression of cyst formation.
Woo, YM. 2015 Disease: ADPKD	MIRA-seq, methylation-sensitive high-resolution melting and validation using EpiTYPER assay.	Renal tissue from ADPKD patients (n = 3) and non-ADPKD healthy renal tissue from renal cell carcinoma patients (n = 3). Urine samples of ADPKD patients evaluated over a period of 21 months (*n* = 53).	Identification of differentially methylated *MUPCDH* as a potential prognostic biomarker of ADPKD.

*Abbreviations*. ^a^*ChIP* chromatin immunoprecipitation, ^b^*H3K4me3* histone three lysine three trimethylation, ^c^*DNA* Deoxyribonucleic acid, ^d^*MSP* methylation specific polymerase chain reaction, ^e^*FSGS* focal segmental glomerulosclerosis. ^f^*ADPKD* autosomal dominant polycystic kidney disease, ^g^*anti-H3K4me2* anti-histone 3 lysine 4 dimethylated, ^h^*DMSO* dimethyl sulfoxide, ^i^*RT-qPCR* quantitative reverse transcription polymerase chain reaction, ^j^*H3K27me3* histone three lysine 27 trimethylation, ^k^*IgAN* IgA nephropathy, ^l^*PBMCs* peripheral blood mononuclear cells, ^m^*H3K9me3* histone three lysine 9 trimethylation, ^n^*MIRA-seq* methylated-CpG island recovery assay

## Discussion

After systematically evaluating current publications relevant to differential methylation in patients with rare renal diseases, this review has identified limited evidence for differential methylation in rare renal diseases. However, what evidence exists is promising and highlights the need for further research to explore differential methylation as a diagnostic and / or prognostic biomarker of rare renal diseases. DNA methylation and renal cancer is reviewed extensively elsewhere and is not discussed in this review [44].

At present, although rare forms of kidney disease significantly affect individuals living with these conditions, there is very poor understanding of the molecular characteristics and best treatment options for these conditions. Even where the underlying genetic cause is known for a rare renal disease there may still be significant unexplained heterogeneity in phenotypes between individuals with the same genotype. Therefore, studying epigenetic features, such as DNA methylation, may offer new insights by providing a mechanism to understand how each individual’s genome interacts with their environment through the epigenome. However, this review highlights that only a small number of studies have been reported researching differential methylation in rare renal diseases:

- IgAN [48–50]
- ADPKD [51–53]
- Rare diseases causing proteinuria including membranous nephropathy [54, 55] and focal segmental glomerulosclerosis (FSGS) [56–59]
- Congenital renal agenesis [60]

This reflects only 5% of the approximated total number of rare renal diseases which exist and thus a significant gap in published research has been identified [4, 5].

IgAN is a condition wherein Immunoglobulin A accumulates in kidney tissues and results in harmful inflammation, which can ultimately lead to end stage renal disease (ESRD) requiring dialysis and renal replacement therapy (RRT). IgAN was a disease included in the RaDaR recruitment criteria and familial IgAN was included in the 100,000 Genomes Project eligibility criteria. In the three studies of IgAN included in this review, DNA methylation was measured through chromatin immunoprecipitation (ChIP) microarray, whole genome microarray analysis of bisulphite converted DNA and methylation specific polymerase chain reaction (MSP). Aberrant methylation was identified as a potential driver in IgAN pathogenesis when identified in CD4+ T cells by causing T helper cell imbalances [49], as a contributor to abnormal glycosylation of IgA1 in IgAN through differential methylation of *Cosmc* [48], and as alterations in the H3K4me3 status identified in IgAN patients along with three significantly differentially methylated candidate genes (*FCRL4, IL1RAPL1* and *PTPRN2*) that may exacerbate IgAN pathology through mediating the cytokine/chemokine cascade and inhibition of protein tyrosine kinase respectively [50].

ADPKD, included in the 100,000 Genomes Project and RaDaR eligibility criteria, is a disease where patients suffer from the growth of cyst formation on the kidney can lead to ESRD and other complications such as kidney stones, polycystic liver disease and brain aneurysms. Our systematic review identified three studies of DNA methylation and ADPKD, where methylation was measured through ChIP and methylated-CpG island recovery assays (MIRA-Seq). These studies identified differential methylation to have a role in ADPKD via upregulation of SMYD2 contributing to renal cyst formation due to methylation of STAT3 and p65, subsequently resulting in increased renal cell proliferation [53], epigenetic silencing of *PKD1* and other ion transport genes in ADPKD due to hypermethylation [52], and identification of reduced expression of *MUPCDH* as a prognostic biomarker of ADPKD [51]. Of interest, the latter two of three studies noted that treatment with methylation inhibitor alleviated cyst formation, thus identifying novel therapeutic targets for ADPKD.

Six of the included articles discussed rare causes of proteinuria, including membranous nephropathy and FSGS (FSGS is included in the RaDaR recruitment criteria). Membranous nephropathy is a rare auto-immune glomerular disease with a global average incidence of 2.5/100,000 individuals [61], characterised by thickening of the glomerular wall and decreased filtration, leading to proteinuria and ultimately loss of kidney function. Our review identified two articles of differential methylation in membranous nephropathy, measured by ChIP sequencing and assays. Increased H3K4me3 was found to exacerbate proteinuria in membranous nephropathy [55], with murine models showing that targeting shRNA against an H3K4 methyltransferase, MLL3, alleviated proteinuria. H3K9me3 alterations were also found to be a biomarker of membranous nephropathy compared to normal control patients [54]. FSGS, included in the RaDaR elgibility criteria, is a condition describing sclerosis of the kidney, with a variety of causes. Three studies of proteinuria featuring FSGS and differential methylation were included, measured by whole genome microarray, MSP and ChIP assays. These studies identified that aberrant methylation in FSGS may cause proteinuria by downregulation of *KLF4*, which has a role in reprogramming somatic cells into induced pluripotent stem (iPS) cells [57]. This was further investigated to show that downregulation of *KLF4* causes nephrin promoter methylation leading to development of proteinuria, which can be alleviated by treatment with an angiotensin receptor blocker (ARB), thus identifying a potential therapeutic target [56]. Interestingly, nephrin was also found to regulate epigenetically in proteinuria caused by FSGS, through aberrant methylation of WHSC1L1-L through interaction with H3K4 and H3K36 [58]. Downregulation of H3K27me3 causing subsequent upregulation of the Notch pathway was found to be associated with albuminuria in glomerular disease, including FSGS [59].

Finally, our comprehensive search identified one study of differential methylation in congenital renal agenesis [60]. Congenital renal agenesis is defined as a condition where one or both kidneys are missing at birth. In this case report of discordant monozygotic twins with congenital renal agenesis, differentially methylated regions are identified but location and whether this is an increase/decrease in methylation is not specified, as well as no discussion of the potential function of these differentially methylated regions.

A number of the studies included in this review investigated methylation in animal models and cell lines, which are imperfect models of human kidney disease [53, 58]. Therefore, it would be helpful to also analyse these candidate differentially methylated genes using DNA isolated from kidney biopsy tissue, saliva, urine or blood in patients with rare renal diseases to assess their utility as diagnostic biomarkers from a minimally invasive test that could be routinely performed in clinic or at a GP surgery; this would minimise risk to patients and improve cost-effectiveness for healthcare professionals.

Despite the scope of this review including literature from 1946, the research identified and described in this review has been conducted within the last decade, reflecting growing awareness of the potential utility of epigenetics in renal disease. Whilst this is encouraging, the studies measured differential methylation in fundamentally different ways and as such comparison between the outcomes were difficult. These different approaches to research methylation were as follows:

- Variations in histone tri-methylation status [50, 54, 55, 58, 59]
- Methyl-transferase activity and impact on signalling pathways [53]
- Hyper/hypo methylation of promotor regions [48, 49, 51, 56–58], and gene bodies [49, 52]

The final objective of this review was to evaluate any research which highlighted the potential for differential methylation as a diagnostic biomarker of rare renal disease. Only two of the studies discussed this potential biomarker development, one being the potential of methylation as a diagnostic biomarker for membranous nephropathy [54] and one the potential for a prognostic biomarker of ADPKD [51]. The focus of the majority of the articles was elucidating pathogenic mechanisms or developing a novel therapies. Therefore, this review highlights that although limited evidence does exist for differential methylation influencing rare renal diseases, further research is required to robustly identify differentially methylated features which could be potential biomarkers and to perform validation studies on such features.

The scarce nature of rare diseases makes it difficult to perform “gold standard” experimental design studies, such as an ethical randomised control trial with strong methodological rigour. One consistent limitation seen across studies included in this review was a lack of accounting for confounding factors in all case-control studies, even though such factors were frequently identified in the discussion and regression analysis may have been appropriate to improve the methodological rigour. This is typical for a developing field, therefore future studies would benefit from standardisation of the detection and analysis of methylation, the inclusion of more robust (laboratory and computational) quality controls, and a comprehensive, transparent reporting structure such as exists for genetic association studies [62].

The wide range of countries from which this research has originated is encouraging since international collaboration is essential to maximise the power of any study given the scarcity of participants with rare renal diseases. Projects such as UK’s 100,000 Genomes Project have left a lasting legacy with ongoing multi-omic analysis (including DNA methylation) currently helping extend understanding and knowledge of rare renal conditions [43]. Additional research would also be helpful to explore if there are significant differences associated with methylation and rare renal diseases between different ethnic groups.

## Conclusions

This review highlights that there has been limited investigation of differential methylation for rare renal diseases, but this limited research is encouraging and will help guide future studies. For example, differential methylation of membranous nephropathy cases compared to healthy controls, significant hypermethylation and under expression of mucin-like protocadherin (*MUPCDH*) between ADPKD kidney tissue and non-ADPKD kidney tissue, differentially methylated regions in congenital renal agenesis, and significantly higher DNA methylation in genes *FCRL4, PTPRN2* and *IL1RAPL1* of IgAN patients compared to healthy controls; all of which highlight DNA methylation as a potential novel biomarker of rare renal disease. Further research focus is required for standardised, international multi-omic analysis of rare renal diseases towards developing a panel of biomarkers with clinical utility. Development of differential methylation diagnostic biomarkers could offer significant aid to patients requiring diagnosis and the health-care professionals struggling to provide diagnosis.

## Additional files

Additional file 1: “PRISMA 2009 checklist.doc” is the PRIMA checklist highlighting where in the submitted manuscript file key components are located. (DOC 63 kb)

Additional file 2: **Table S1**. MEDLINE via Ovid search terms adapted for use in other databases. **Table S2**. Quality appraisal and data extraction template form for case-control studies. **Table S3**. Quality appraisal and data extraction form for case report studies. **Table S4**. Study characteristics of articles included in the review (DOCX 49 kb)

## Data Availability

Data is primarily derived from peer-review publications in the public domain, which may be subject to copyright. All data generated or analysed during this study are included in this published article [and its Additional files].

## Abbreviations

ADPKD: Autosomal Dominant Polycystic Kidney Disease
IgAN: IgA Nephropathy
MUPCDH: Mucin-like protocadherin
PKD: Polycystic Kidney Disease
RaDaR: Registry of Rare Kidney Disorders
SNPs: Single Nucleotide Polymorphisms
