# Supplementary material for: Differential methylation as a diagnostic biomarker of rare renal diseases: a systematic review
Source: BMC Nephrol. 2019 Aug 16;20:320. doi: 10.1186/s12882-019-1517-5 (PMC6697952; doi:10.1186/s12882-019-1517-5)
Supplement: Supplementary file 2 — Table S1. MEDLINE via Ovid search terms adapted for use in other databases. Table S2. Quality appraisal and data extraction template form for case-control studies. Table S3. Quality appraisal and data extraction form for case report studies. Table S4. Study characteristics of articles included in the review (DOCX 49 kb) [file 12882_2019_1517_MOESM2_ESM.docx]

**Differential methylation as a diagnostic biomarker of rare renal diseases: a systematic review.**

Kerr, K., McAneney, H., Flanagan, C., Maxwell, A.P., & McKnight, A.J.

**SUPPLEMENTARY MATERIALS AND METHODS**

Table S1. MEDLINE via Ovid search terms adapted for use in other databases.

| **#** | **Search terms** |
| --- | --- |
|  | (exp Methylation/ OR Methylat*) |
|  | ((Alport OR Bartter OR Gitelman OR berger* OR Liddle OR lowe OR Oculocerebrorenal) ADJ3 (syndrome OR disease*)) |
|  | ((Atypical OR Nonenteropath*) ADJ3 Hemolytic ADJ3 Uremi*) |
|  | ((cyst* OR polycyst*) ADJ3 (kidney* OR renal*)) |
|  | ((familial OR Hereditary) ADJ3 (Nephritis OR haematuria OR hematuria)) |
|  | ((IgA OR Immunoglobulin-A) ADJ3 (Nephropath* OR Nephrit*)) |
|  | ((kidney* OR renal) ADJ3 (stone* OR calculi* OR calcificat*)) |
|  | ((Membranoproliferative OR Hypocomplement* OR Mesangiocapillar*) ADJ3 (glomerulonephrit*)) |
|  | ((Nonenteropathic OR Non-Stx) ADJ3 HUS) |
|  | ((proteinuric ADJ3 (kidney OR renal)) |
|  | ((Renal OR kidney) ADJ3 Tubul* ADJ3 Acidosis) |
|  | ((Transphosphoribosidase OR Phosphoribosyltransferase OR APRTase) ADJ3 Deficien*) |
|  | (C3 ADJ3 (Glomerulonephrit* OR Glomerulopath*)) |
|  | (Cerebr* ADJ3 Ocul* ADJ3 (Renal OR kidney*)) |
|  | (Cystine ADJ3 (defect* OR Disease* OR Diathes*)) |
|  | (Dense ADJ3 Deposit ADJ3 Disease) |
|  | (Diffuse ADJ3 mesangial ADJ3 sclerosis) |
|  | (extreme ADJ3 early ADJ3 hypertens*) |
|  | (familial ADJ3 steroid ADJ3 nephrotic) |
|  | (focal ADJ3 (glomeruloscleros* OR Glomerulonephrit*)) |
|  | (foot ADJ3 process ADJ3 effacement) |
|  | (glomerular ADJ3 basement ADJ3 nephropath*) |
|  | (Hyperuric* ADJ3 Nephropath*) |
|  | (membranous ADJ3 glomeruloneph*) |
|  | (Pregnan* ADJ3 Chronic* ADJ3 (renal OR Kidney*))).mp.) |
|  | Acidosis, Renal Tubular/ |
|  | Adenine Phosphoribosyltransferase/ |
|  | Atypical Hemolytic Uremic Syndrome/ |
|  | Bartter Syndrome/ |
|  | CAKUT.mp. |
|  | CAKUT.nm |
|  | cystinosis/ |
|  | Cystinuria/ |
|  | Gitelman Syndrome/ |
|  | Glomerulonephritis, IGA/ |
|  | Glomerulonephritis, Membranoproliferative/ |
|  | Glomerulonephritis, Membranous/ |
|  | Glomerulosclerosis, Focal Segmental/ |
|  | Kidney Diseases, Cystic/ |
|  | Liddle Syndrome/ |
|  | Mesangial sclerosis, diffuse.nm. |
|  | Nephritis, Hereditary/ |
|  | Nephrocalcinos* |
|  | Nephrocalcinosis/ |
|  | Nephrolithiasis/ |
|  | Oculocerebrorenal Syndrome/ |
|  | Polycystic Kidney, Autosomal Dominant/ |
|  | Polycystic Kidney, Autosomal Recessive/ |
|  | Pseudoaldosteron* |
|  | (2 OR 3 OR 4 OR 5 OR 6 OR 7 OR 8 OR 9 OR 10 OR 11 OR 12 OR 13 OR 14 OR 15 OR 16 OR 17 OR 18 OR 19 OR 20 OR 21 OR 22 OR 23 OR 24 OR 25 OR 26 OR 27 OR 28 OR 29 OR 30 OR 31 OR 32 OR 33 OR 34 OR 35 OR 36 OR 37 OR 38 OR 39 OR 40 OR 41 OR 42 OR 43 OR 44 OR 45 OR 46 OR 47 OR 48 OR 49) |
|  | 1 OR 50 |

Key: exp = subject heading tree has been exploded and incorporates all narrow headings of its tree structure. / = all subheadings have been searched within the subject heading. .mp. = multi-purpose, meaning title, abstract and subject heading have been searched. * = Any group of characters, including no characters. ADJ(n) = Adjacent within a specified (n) number of words.

Table S2. Quality Appraisal and Data Extraction Template Form for Case Control Studies

|  |  | Yes | No | Unclear | Not Applicable | Comments |
| --- | --- | --- | --- | --- | --- | --- |
| 1 | Was the primary focus of the paper methylation? |  |  |  |  |  |
| 2 | Were the groups comparable other than the presence of disease in cases or the absence of disease in controls? |  |  |  |  |  |
| 3 | Were cases and controls matched appropriately? |  |  |  |  |  |
| 4 | Was methylation measured in in a standard reliable way? |  |  |  |  |  |
| 5 | Was methylation measured in the same way for cases and controls? |  |  |  |  |  |
| 6 | Were confounding factors identified? |  |  |  |  |  |
| 7 | Were strategies to deal with confounding factors identified? |  |  |  |  |  |
| 8 | Was the exposure period of interest long enough to be meaningful? |  |  |  |  |  |
| 9 | What statistical analysis was used and was this analysis appropriate? |  |  |  |  |  |
| 10 | Were experimental controls used? |  |  |  |  |  |

Table S3. Quality Appraisal and Data Extraction Form for Case Report Studies

|  | | Yes | No | Unclear | Not Applicable | Comments |
| --- | --- | --- | --- | --- | --- | --- |
| 1 | Was the primary focus of the paper methylation? |  |  |  |  |  |
| 2 | Was the patient’s history described clearly? |  |  |  |  |  |
| 3 | Was the patient’s current clinical condition described clearly? |  |  |  |  |  |
| 4 | Was methylation measured in in a standard reliable way? |  |  |  |  |  |
| 5 | Were confounding factors identified? |  |  |  |  |  |
| 6 | Were strategies to deal with confounding factors identified? |  |  |  |  |  |
| 7 | Were experimental controls used? |  |  |  |  |  |

| Table S4: Study characteristics of articles included in the review. | | | |
| --- | --- | --- | --- |
| **First Author, Year of Publication, Title and DOI** | **Research Objective** | **Design Information** | **Relevant Results** |
| Fujino, Takayuki. 2016.  DOI:  10.1186/s12882-016-0390-8  Number in reference list: 55 | To investigate the role of H3K4me3*^a^* in nephrotic syndrome caused by MN*^b^* | Observational design: case control  Population:  Renal biopsies from patients diagnosed with nephrotic syndrome caused by MN (n=6). Control participants with other causes of nephrotic syndrome; minimal change disease (n=2), FSGS*^c^* (n=2) and amyloidosis (n=3). Also renal biopsies from participants (n=3) with no glomerulonephropathy (Control A) and an age matched healthy control tissue section (Control B). Male murine models of proteinuria.  Methylation measurement:  Immunohistochemical staining of H3K4me3 localisation in glomeruli of mice and human participants. For murine models western blotting was followed by acid extraction followed by ELISA*^d^* and ChIP*^e^* assays to determine H3K4me3 localisation and expression levels.  Methodological rigour: Weak | Significantly higher overlapping expression of H3K4me3 and cathepsin L in patients with MN compared to control A and B participants, as well as compared to control participants with other causes of nephrotic syndrome. H3K4me3 staining intensity was positively correlated with proteinuria in primary MN patients. Synaptopodin expression levels were inversely correlated with H3K4me3 expression and proteinuria levels.  Mice with induced proteinuria showed elevated H3K4me3 levels compared to controls, with reduced synaptopodin and increased cathepsin L levels.  Upregulation of H3K4me3 and indeed cathepsin L was inhibited by downregulation of the methyltransferase MLL3. Use of the MLL3 shRNA also significantly restored synaptopodin levels. This in turn reduced proteinuria levels and improved renal function. Similar results were seen in cultured podocytes. |
| Hayashi, K. 2014.  KLF4-dependent epigenetic remodeling modulates podocyte phenotypes and attenuates proteinuria.  DOI: 10.1172/jci69557  Number in reference list: 57 | To investigate the expression levels of KLF4 in kidney podocytes, identifying potential novel therapeutic targets in proteinuria. | Observational design: case control  Population:  Murine models of proteinuria including FSGS (n=11) diabetic nephropathy (n=9), minimal change disease (n=10) and normal controls (n=9). Human renal biopsies from the same proteinuric diseases.  Methylation measurement:  Microarray-based genome wide DNA*^f^* methylation profiling system. These results were confirmed using MSP*^g^* and bisulphite sequencing.  Methodological rigour: weak | *KLF4* expression is decreased in ADM*^h^* treated murine models and in patients with proteinuria compared to healthy controls.  Significantly increased demethylation of nephrin promoter CpG methylation in *KLF4* overexpressing cells.  Treatment of *Klf4* knock out mice with ADM caused a significant increase in nephrin promoter methylation, however no significant difference in nephrin methylation of knock out mice was seen in the absence of ADM treatment. |
| Hayashi, K. 2015.  Renin-angiotensin blockade resets podocyte epigenome through Kruppel-like Factor 4 and attenuates proteinuria.  DOI: 10.1038/ki.2015.178  Number in reference list: 56 | To investigate the use of ARB^i^ to attenuate proteinuria through KLF4 epigenetic remodelling. | Observational design: case control  Population:  Murine models of ADM nephropathy, (n=5 in each treatment group). Samples from patients with proteinuric glomerular diseases including minimal change disease (n=9), FSGS (n=8), diabetic nephropathy (n=8) and normal controls (n=8). Immortal human podocyte cell lines.  Methylation measurement:  Bisulphite treatment of DNA followed by MSP.  Methodological rigour: weak | ARB treatment of murine proteinuria models resulted in increased *KLF4* expression, decreased nephrin promoter methylation and a significant reduction in albuminuria.  *KLF4* knock out mice have reduced attenuated proteinuria from the ARB treatment than ADM controls. More severe nephrin promoter methylation was seen in knock out mice.  Angiotensin II treatment of human podocyte cell lines reduced *KLF4* expression and increased nephrin promoter methylation. |
| Ito, Y. 2017.  Wolf-Hirschhorn syndrome candidate 1-like 1 epigenetically regulates nephrin gene expression.  DOI: 10.1152/ajprenal.00305.2016  Number in reference list: 58 | To investigate the role of the two isoforms, long and short, of the *WHSC1L1 gene* as epigenetic modifiers of nephrin gene regulation, to understand the implications for congenital and acquired nephrotic syndrome. | Observational design: case control  Population:  Murine models of proteinuria, *HEK^j^* cell lines, zebrafish models of proteinuria.  Methylation measurement:  ChIP assays were used to analyse the levels of histone methylation of HEK cells expressing *WHSC1L1* long form compared to the same cell line not expressing *WHSC1L1* long form*.*  Methodological rigour: weak | *WHSC1L1* long form has methyltransferase activity associated with H3K4 and H3K36.  RNA silencing with *WHSC1L1* increases nephrin expression.  In vivo H3K4 trimethylation was reduced in HEK293-WHSC1L1 long form cells compared to regions in HEK-293 cells.  Following induction of proteinuria in murine models, *WHSC1L1-L* expression levels decrease and nephrin expression levels increase. |
| Jin, M. 2014  Genomic and epigenomic analyses of monozygotic twins discordant for congenital renal agenesis.  DOI: 10.1053/j.ajkd.2014.01.423  Number in reference list: 59 | To assess the contributions of genetics and epigenetics in congenital renal agenesis. | Observational design: case report  Population information:  Chinese female, 30 years of age, left kidney renal agenesis, with a monozygotic twin without renal agenesis, no family history of renal agenesis and no current clinical symptoms.  Methylation measurement:  Differentially methylated regions were measured by reduced-representation bisulphite sequencing.  Methodological rigour: weak | No significant differences at the genomic level either in single nucleotide variation or copy number variation.  514 differentially methylated regions detected between patient with renal agenesis and patient without. |
| Li, LX. 2017  Lysine methyltransferase SMYD2 promotes cyst growth in autosomal dominant polycystic kidney disease.  DOI: 10.1172/JCI90921  Number in reference list: 53 | To investigate the role of SMYD2, a lysine methyltransferase protein, in renal cyst growth in patients with ADPKD*^k^*. | Observational design: case control  Population information:  Double conditional knockout of *Pkd1* and *Smyd2* in murine models of ADPKD (n=12) compared to single knockout of *Pkd1* (n=14). Murine models (n=12) of ADPKD (*Pkd1* knockouts) treated with AZ505 compared to DMSO*^l^* injected controls (n=12) and conditional *Pkd1* knockouts (n=14) compared to DMSO injected controls (n=14). Human ADPKD cells were also utilised and compared to normal kidney cells.  Methylation measurement:  ChIP with anti-H3K4me2*^m^* antibodies and anti-SMYD2 antibodies. Methylation sites localised using a flag-tagged protein.  Methodological rigour: weak | SMYD2 protein levels and mRNA*^n^* expression increased in Pkd1 knock out mouse kidney cells and human ADPKD cells compared to wildtypes.  Cyst formation significantly reduced in the Smyd2 knock out mice. Double conditional knockout mice of Smyd2 and Pkd1 lived to an average of 22.2 days with delayed cyst formation whilst Smyd2 positive mice died of polycystic kidney disease at an average of 16.3 days, significantly less.  Methylation of STAT3 and p65 (components of NF-kB) increased in Pkd1-null MEK*^o^* cells compared to wildtype. This was decreased following inhibition of SMYD2 with AZ505.  Inhibition of SMYD2 with the AZ505 inhibitor and silencing of Smyd2 in MEK cells decreased the methylation status of H3K4 and H3K36.  SMYD2 interacted with p53 and an increase in methylation of p53 was seen in Pkd1-null MEK cells compared to wildtype MEK cells. |
| Majumder, Syamantak. 2018.  Shifts in podocyte histone H3K27me3 regulate mouse and human glomerular disease.  DOI: 10.1172/JCI95946  Number in reference list: 59 | Investigation of H3K27me3*^p^* regulation in human glomerular disease. | Observational design: case control.  Population:  Murine models of FSGS-like glomerular injury. Tissue samples of human participants with the glomerular diseases diabetic glomerulosclerosis (n=12) compared to age matched healthy individuals (n=12) and FSGS (n=10) compared to implantation biopsy tissue (n=9) taken at the time of kidney transplantation (none with diabetes).  Methylation measurement:  Immunohistochemical staining of H3K27me3 in mouse and human kidney sections, RT-qPCR*^q^* and ChIP assays with H3K27me3 antibody.  Methodological rigour: weak | Reduced H3K27me3 levels and increased albuminuria was seen in mice with knockouts of the trimethylating enzyme EZH2 compared to controls. Heightened levels of Notch pathway components *N1-ICD* and *Hey1* were also seen in knockout mice.  In cultured mice podocytes, loss of H3K27me3 was associated with increased Notch ligand *Jagged-1* levels and glomerular disease when treated with EPZ-6438 and EZH2 short hairpin RNA inhibition.  Increasing H3K27me3 levels with the selective inhibition of *Jmjd3, UTX* and *GSK-J4* decreased Jagged-1 levels, attenuated the decreased in podocin levels and decreased albuminuria levels in adriamycin treated mice.  Immunostaining showed a loss of H3K27me3 and increased *Jagged-1* and *UTX* expression in patients with glomerular disease compared to controls. Unlike in mice, loss of *EZH2* did not associate with loss of H3K27me3.  In mice with previously diminished glomeruli and loss of H3K27me3, analogous to human glomerular disease samples, inhibition with *GSK-J4* treatment prevented further loss of H3K27me3 and podocytes, attenuating albuminuria progression. |
| Qi, S. 2012  CpG Array Analysis of Histone H3 Lysine 4 Trimethylation by Chromatin Immunoprecipitation Linked to Microarrays Analysis in Peripheral Blood Mononuclear Cells of IgA Nephropathy Patients.  DOI: 10.3349/ymj.2012.53.2.377  Number in reference list: 50 | To elucidate variations in H3K4me3 and DNA methylation levels of patients with IgAN*^r^* | Observational design: case control  Population information:  Patients of the 181th Hospital of Guangzhou Military Area with IgAN. Of these patients, the average age was 32.56 years (n=15) and in sex-matched healthy controls 34.06 years (n=15).  Methylation measurement:  ChIP microarray and real time quantitative MSP.  Methodological rigour: weak | Of the four candidate H3K4me3 genes selected from the ChIP-chip analysis, two genes displayed an increase in H3K4me3 (*FCRL4* and *GALK2*) and two displayed a decrease (*PTPRN2* and *IL1RAPL1*) significantly different between IgAN patients and controls.  mRNA expression was reduced in one of these H3K4me3 candidate genes, *PTPRN2*, whilst the other three displayed increased mRNA expression compared to healthy controls.  DNA methylation levels were significantly higher in *FCRL4, PTPRN2* and *IL1RAPL1* methylation levels in IgAN patients compared to healthy controls, but no significant difference was found in *GALK2.* |
| Sallustio, F. 2016  Aberrantly methylated DNA regions lead to low activation of CD4+ T-cells in IgA nephropathy.  DOI: 10.1042/CS20150711  Number in reference list:  49 | To investigate the role of differential methylation of genes involved T-Cell signalling and the subsequent differences in expression and T-Cell proliferation. | Observational design: case control  Population information: Renal biopsies from IgAN patients (n=24) and healthy subjects from South Italy (n=24). This population was age matched.  Methylation measurement:  Whole genome methylation sequencing of CD4+ T cells. The most significantly differentially methylated sites were validated by pyrosequencing to identify hyper and hypomethylated regions.  Methodological rigour: weak | Following whole genome methylation sequencing on CD4+ T-Cells the most significant differentially methylated regions were genes involved in T-cell signalling (*DUSP3, TRIM27* and *VTRNA2-1*).  *DUSP3* and *TRIM27* were hypomethylated and upregulated in IgAN patients and *VTRNA2-l* was hypermethylated and downregulated in IgAN patients.  Treatment of CD4+ T-cells with a DNA methyltransferase inhibitor (5-aza-2’-deoxycytidine) caused reduced methylation in all three of the above genes (92% to 21.5% in *DUSP3*, 37.9% to 28.3% in *TRIM27* and 26.2% to 17.2% in *VTRNA2-1)*.  CD4+ T-cells show impaired proliferation (1.8-fold lower) in IgAN cases compared to controls.  IL-2/IL-5 ratio significantly higher in IgAN patients, reflecting a shift towards Th1-like cells. |
| Sui, WG. 2014  ChIP-seq analysis of histone H3K9 trimethylation in peripheral blood mononuclear cells of membranous nephropathy patients.  DOI: 10.1590/1414-431X20132809  Number in reference list: 54 | To analyse alterations in H3K9me3*^s^* of membranous nephropathy patients to further elucidate the pathogenesis of the disease and identify potential biomarkers. | Observational design: case control  Population information:  Patients of the 181st hospital diagnosed with membranous nephropathy (n=10). Healthy controls (n=10), sex and race matched. Average age of patients was 39.50 years and 37.89 in healthy controls.  Methylation measurement:  ChIP-sequencing of H3K9me3 followed by Model-based Analysis of ChIP-sequencing which identified enriched H3K9me3 peaks.  Methodological rigour: weak | Significant differential H3K9 methylation was seen in 108 genes of membranous nephropathy compared to healthy control (75 showed increased expression and 33 showed decreased expression).  Of the selected five candidate genes with the greatest differences compared to healthy controls;  -*DGCR6* had increased H3K9 methylation  -*SNX16* had increased expression  -*CNTN4* had increased expression  -*BIRC2* and *BIRC3* had decreased H3K9 methylation |
| Sun, Q. 2015.  DNA methylation in *Cosmc* promoter region and aberrantly glycosylated IgA1 associated with pediatric IgA nephropathy  DOI: 10.1371/journal.pone.0112305  Number in reference list: 48 | To investigate if CpG promoter methylation of the gene *Cosmc* is responsible for its decreased expression and higher levels of aberrantly glycosylated IgA1 in patients with IgAN compared to healthy controls. | Observational design: case control  Population information:  Three paediatric populations: (I) IgAN patients (n=26) with a mean age of 9.93, (II) other renal disease patients (n=11) including Alport syndrome, mild mesangial proliferative glomerulonephritis, thin basement nephropathy and membranous nephropathy with mean age of 8.8, (III) healthy controls (n=13), mean age 12.12.  Methylation measurement:  Bisulphite treatment of DNA followed by MSP of *Cosmc* gene promoter regions.  Methodological rigour: weak | IL-4 treated media of IgAN lymphocytes showed significantly higher methylation compared to either of the control groups.  No significant difference in *Cosmc* methylation was seen in control populations in plain media or either of the drug treated media.  For all three populations, IL-4 increased *Cosmc* methylation and 5-Aza-2'-deoxycytidine decreased *Cosmc* methylation.  IL-4 induced increases in *Cosmc* methylation were most pronounced in IgAN patients and less decreases were seen in IgAN lymphocyte methylation following 5-Aza-2'-deoxycytidine treatment compared to the two control populations.    A negative linear correlation was seen between IgAN lymphocyte methylation and mRNA expression in all populations. A positive correlation was seen between aberrant glycosylation of IgA1 and *Cosmc* promoter methylation. |
| Woo, YM. 2014  Genome‑wide methylation profiling of ADPKD identified epigenetically regulated genes associated with renal cyst development  DOI: 10.1007/s00439-013-1378-0  Number in reference list: 52 | To investigate the role of epigenetic alterations in ADPKD cyst development. | Observational design: case control  Population information:  Cystic renal cortex samples from ADPKD patients (n=3) and non-ADPKD samples from renal cell carcinoma patients (n=3) used as a normal control. Madin-Darby Canine Kidney cells also used.  Methylation measurement:  Differential methylation was measured at a whole genome level by MIRA-seq*^u^* giving methylation levels for every 200-bp interval and a methylation enrichment score was calculated. Histone methylation status was measured by Histone ChIP-qPCR.  Methodological rigour: weak | Hypermethylation accounted for 91% of differential methylation compared to only 9% hypomethylation. Of the differentially methylated fragments, this did not include promoter regions (accounted for 0% hypermethylation and 1% hypomethylation).  A 5.93-fold higher level of hypermethylation was seen in the in exonic regions of ADPKD patients.  *PKD1* was found to be hypermethylated in ADPKD patients, specifically in the gene-body, along with several other genes with functions affecting cystogenesis.  Treatment with methylation inhibitors repressed DNA methylation of *Pkd1* and cyst formation simultaneously whilst increasing histone modifications by increased DNA methylation at the *Pkd1* gene body region. |
| Woo, YM. 2015  Epigenetic silencing of the *MUPCDH* gene as a possible prognostic biomarker for cyst growth in ADPKD.  doi: 10.1038/srep15238  Number in reference list: 51 | To investigate if differential methylation of *MUPCDH* can be used as a prognostic biomarker of ADPKD | Observational design: case control  Population information:  Renal tissue from ADPKD patients (n=3) and non-ADPKD healthy renal tissue from renal cell carcinoma patients (n=3). Urine samples of ADPKD patients evaluated over a period of 21 months (n=53).  Methylation measurement:  Methylation was measured by MIRA-seq and *MUPCDH* promoter region methylated was validated using methylation-sensitive high-resolution melting. Further validation was performed using the EpiTYPER assay.  Methodological Rigour: weak | *MUPCDH* was significantly hypermethylated and under expressed in the promoter region compared to ADPKD and non-ADPKD kidney tissues.  Following in vitro treatment with a demethylating agent, *MUPCDH* methylation decreased, mRNA expression increased, and protein level was restored in ADPKD tissue.  There was a significant difference in kidney volume of ADPKD patients in the 100% methylated group compared to the 0-75% methylated group annually, however no significant difference was seen in baseline kidney function. |

**Abbreviations:** ^a^H3K4me3: histone 3 lysine 4 trimethylated, ^b^MN: membranous nephropathy, ^c^FSGS: Focal segmental glomerulosclerosis, ^d^ELISA: enzyme-linked immunosorbent assay, ^e^ChIP: chromatin immunoprecipitation assay, ^f^DNA: deoxyribonucleic acid, *^g^*MSP: methylation specific polymerase chain reaction, ^h^ADM: adriamycin, ^i^ARB: angiotensin receptor blocker, ^j^HEK: human embryonic kidney, ^k^ADPKD: autosomal dominant polycystic kidney disease, ^l^DMSO: dimethyl sulfoxide, ^m^anti-H3K4me2: anti histone 3 lysine 4 dimethylated, *^n^*mRNA: messenger ribonucleic acid, ^o^MEK: mouse embryonic kidney, ^p^H3K27me3: histone 3 lysine 27 trimethylated, ^q^RT-qPCR: reverse transcriptase quantitative polymerase chain reaction, ^r^IgAN: IgA nephropathy, ^s^H3K9me3: histone 3 lysine 9 trimethylated, ^t^MIRA-seq: methylated-CpG island recovery assay.
